# Metabolic Dysfunction-Associated Fatty Liver Disease (MAFLD) Is Associated with Cervical Stromal Involvement in Endometrial Cancer Patients: A Cross-Sectional Study in South China

**DOI:** 10.3390/curroncol30040287

**Published:** 2023-03-29

**Authors:** Xite Lin, Chunxia Chen, Tingting Jiang, Jincheng Ma, Lixiang Huang, Leyi Huang, Huifang Lei, Yao Tong, Guanxiang Huang, Xiaodan Mao, Pengming Sun

**Affiliations:** 1 Laboratory of Gynecologic Oncology, Fujian Maternity and Child Health Hospital, College of Clinical Medicine for Obstetrics & Gynecology and Pediatrics, Fujian Medical University, Fuzhou 350001, China; 2 Fujian Key Laboratory of Women and Children’s Critical Diseases Research, Fujian Maternity and Child Health Hospital, Fuzhou 350001, China; 3 Fujian Clinical Research Center for Gynecological Oncology, Fujian Maternity and Child Health Hospital, Fuzhou 350001, China; 4 Department of Imaging, Fujian Maternity and Child Health Hospital, Affiliated Hospital of Fujian Medical University, Fuzhou 350001, China

**Keywords:** metabolic dysfunction-associated fatty liver disease, endometrial cancer, cervical stromal involvement, hepatic steatosis index

## Abstract

Background: Metabolic dysfunction-associated fatty liver disease (MAFLD) is a significant health issue closely associated with multiple extrahepatic cancers. The association between MAFLD and clinical outcomes of endometrial cancer (EC) remains unknown. Methods: We retrospectively included 725 EC patients between January 2012 and December 2020. The odds ratios (ORs) were calculated using logistic regression analyses. Kaplan–Meier survival curves were used for survival analysis. Results: Among EC patients, the prevalence of MAFLD was 27.7% (201/725, 95% confidence interval (Cl) = 0.245–0.311). MAFLD was significantly associated with cervical stromal involvement (CSI) (OR = 1.974, 95% confidence interval (Cl) = 1.065–3.659, *p* = 0.031). There was a significant correlation between overall survival (OS) and CSI (HR = 0.31; 95%CI: 0.12–0.83; *p* = 0.020), while patients with MAFLD had a similar OS to those without MAFLD (*p* = 0.952). Moreover, MAFLD was significantly associated with CSI in the type I EC subgroup (OR = 2.092, 95% confidence interval (Cl) = 1.060–4.129, *p* = 0.033), but not in the type II EC subgroup (*p* = 0.838). Further logistic regression analysis suggested that the hepatic steatosis index (HSI) was significantly associated with CSI among type I EC patients without type 2 diabetes mellitus (T2DM) (OR = 1.079, 95% confidence interval (Cl) = 1.020–1.139, *p* = 0.012). Conclusions: About one-quarter of our cohort had MAFLD. MAFLD was associated with the risk of CSI in EC patients, and this association existed in type I EC patients but not in type II EC patients. Furthermore, the HSI can help predict CSI in type I EC patients without T2DM.

## 1. Introduction

Endometrial cancer (EC) was the most common gynecologic malignancy and the sixth most common cancer among women worldwide in 2020 [1]. Several studies have shown that obesity and conditions associated with metabolic syndrome (MetS) are risk factors for the development of EC [2,3]. Of the 20 most common obesity-related tumor types, EC has the strongest link with obesity [4,5]. As a result of adiposity affecting the synthesis and bioavailability of endogenous sex steroid hormones, the majority of factors associated with EC are associated with high estrogen levels [6]. Traditionally, EC is mainly categorized as type I and type II [7]. Type I ECs are estrogen-dependent and well-differentiated adenocarcinomas that are more closely associated with obesity and have a good prognosis. Type II ECs are non-estrogen-related, poorly differentiated tumors that behave in an aggressive manner [8].

EC is typically diagnosed when the tumor is confined to the uterine corpus, in which case the EC patient usually has a good prognosis, and in a small number of cases, cancer also develops in the cervical stroma (International Federation of Gynecology and Obstetrics (FIGO) stage II). Cervical stromal involvement (CSI) is considered to be an unfavorable risk factor for recurrence [9,10]. In addition, CSI is thought to increase the risk of lymph node metastasis [11]. For these patients, radical hysterectomy, including parametrectomy and lymphadenectomy, may be recommended, and postoperative adjuvant therapy is indicated to reduce the risk of recurrence [9,12]. As EC cannot be screened for with a standardized test, prevention based on health behaviors (such as obesity, weight gain, metabolic syndrome and diabetes prevention) is feasible [13]. Therefore, preoperative identification of patients at high risk of CSI is important both in terms of treatment and prognosis.

Metabolic-associated fatty liver disease (MAFLD), formerly named nonalcoholic fatty liver disease (NAFLD), is an emerging public health concern worldwide [14]. The pathogenic mechanisms underlying MAFLD are thought to be related to potentiated metabolic dysfunctions [15]. There is epidemiological evidence that MAFLD is related to obesity, MetS and type 2 diabetes mellitus (T2DM) [16], and its pathogenesis is closely entangled with increased adiposity, dyslipidemia and insulin resistance (IR) [17]. As previously pointed out, EC and MAFLD share common risk factors such as obesity, MetS, and T2DM. Nevertheless, there is still debate on the relationship between EC and MAFLD. Numerous studies have revealed a link between NAFLD/MAFLD and an elevated risk of extrahepatic malignancies, particularly gastrointestinal cancers, breast cancer, and gynecological cancers [18,19,20,21]. Furthermore, NAFLD/MAFLD was found to be an independent risk factor for mortality from any type of malignancy in a T2DM cohort study [22]. Accordingly, both MAFLD and NAFLD tend to be related to metabolic dysregulation-related events and an increased risk of obesity-related cancer [23].

The liver is the main organ of glucose and lipid metabolism. There is an obvious link between MAFLD and obesity, both of which can cause abnormal lipid metabolism in the liver [24]. The hepatic steatosis index (HSI) is one of the obesity and lipid-related indices that has good performance in non-invasive screening for NAFLD/MAFLD [25]. HSI, encompassing sex, alanine aminotransferase (ALT), aspartate aminotransferase (AST), body mass index (BMI) and diabetic status, is also strongly linked to insulin resistance and metabolic syndrome [26].

However, there has not yet been a study reporting MAFLD in the context of the clinical outcomes of EC. Therefore, the aim of this study was to investigate the associations of MAFLD on the risk of CSI among EC patients.

## 2. Materials and Methods

### 2.1. Study Population and Selection of Patients for Analysis

Our study was a retrospective analytic cross-sectional study. We retrospectively evaluated EC patients who underwent total hysterectomy and bilateral salpingo-oophorectomy with or without lymphadenectomy at Fujian Maternity and Child Health Hospital, Affiliated Hospital of Fujian Medical University, from January 2012 to December 2020.

**Figure 1:**
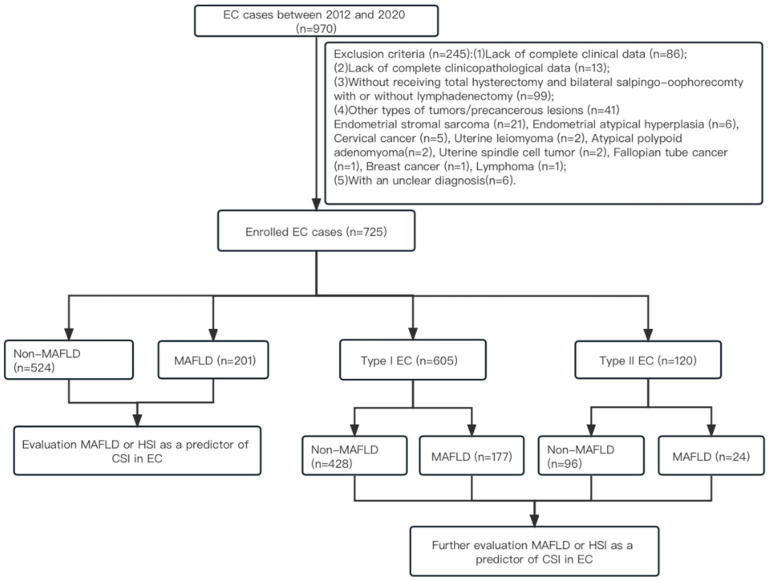
Flowchart of study. Abbreviations: EC: endometrial cancer; HSI: hepatic steatosis index; MAFLD: metabolic dysfunction-associated fatty liver disease; CSI: cervical stromal involvement.

The exclusion criteria included the following: (1) had a lack of complete clinical data (*n* = 86); (2) had a lack of complete clinicopathological data (*n* = 13); (3) did not undergo total hysterectomy and bilateral salpingo–oophorectomy with or without lymphadenectomy (*n* = 99); (4) had other types of tumors/precancerous lesions (*n* = 41): endometrial stromal sarcoma (*n* = 21), endometrial atypical hyperplasia (*n* = 6), cervical cancer (*n* = 5), uterine leiomyoma (*n* = 2), atypical polypoid adenomyoma (*n* = 2), uterine spindle cell tumor (*n* = 2), fallopian tube cancer (*n* = 1), breast cancer (*n* = 1), lymphoma (*n* = 1); and (5) had an unclear diagnosis (*n* = 6). According to exclusion criteria, 725 cases with EC were finally included in the current study (Figure 1). This retrospective study was approved by the Fujian Maternity and Child Health Hospital Ethics commission (approval number 2022KYLLR03028), and informed consent was waived (2023KYLLRK01088).

### 2.2. Data Collection

Data on demographic and clinical indicators were obtained from patient medical records provided by our institution’s biochemical database. Upon admission to the hospital, height and weight were measured. Body mass index (BMI), which is computed as weight (kg)/height (m)^2^, was then determined. Normal weight is defined as having a BMI of 18.5–22.9 kg/m^2^, and overweight is defined as having a BMI of 24.0–24.9 kg/m^2^. A BMI of more than 25.0 kg/m^2^ was used to categorize people as obese [27]. We staged patients according to FIGO (International Federation of Gynecology and Obstetrics) guidelines. The pathological diagnosis of the EC samples was made by two specialized gynecological pathologists based on the Bokhman classification for type I/II EC in a double-blinded manner [7]. Type I endometrial cancer was defined as grade 1–2 endometrioid adenocarcinomas, whereas type II endometrial cancer was defined as grade 3 endometrioid, serous, clear cell, undifferentiated, carcinosarcoma, squamous and adenosquamous histology types [28]. For the mixed histology type, patients with high expression of ER and PR were classified as type I EC, whereas other types were defined as type II EC. The expression of ER and PR was evaluated by immunohistochemistry. ER/PR low expression included ER/PR (−/±); ER/PR high expression included ER/PR (+/++/+++). Pathology results, including histological grade, cervical stromal involvement (CSI), myometrial infiltration (MI), lymphovascular space invasion (LVSI) and lymph node metastasis (LNM), were determined by a postoperative pathology report. Blood testing was carried out within 1 week before surgery.

### 2.3. Diagnosis of MAFLD

International specialists came to an agreement that MAFLD was defined as having any one of the following three characteristics in addition to hepatic steatosis/fatty liver as determined by abdominal ultrasonography: (1) being overweight or obese (BMI ≥ 23 kg/m^2^; depending on the Asian criteria); (2) having T2DM; or (3) having at least two metabolic abnormalities [29]. Metabolic abnormality criteria included (1) central obesity (waist circumference ≥ 90 cm in Asian male, ≥80 cm in Asian female), (2) prediabetes (fasting glucose levels 5.6 to 6.9 mmol/L or HbA1c 5.7–6.4%), (3) blood pressure ≥ 130/85 mmHg or specific drug treatment, (4) plasma triglycerides ≥ 1.70 mmol/L or treated for dyslipidemia, (5) plasma HDL cholesterol 1.0 mmol/L for men and <1.3 mmol/L for women or specific drug treatment, (6) homeostasis model assessment and insulin resistance (HOMA-IR) ≥ 2.5, (7) plasma high-sensitivity C-reactive protein level >2 mg/L. HOMA-IR was calculated according to the equation HOMA-IR = fasting insulin in μU/mL × fasting glucose in mmol/L divided by 22.5. The MAFLD criteria include waist circumference, HOMA-IR and plasma high-sensitivity C-reactive protein levels, but these variables were not included in our dataset.

### 2.4. Definition of Hepatic Steatosis Index (HSI)

The hepatic steatosis index (HSI) has been reported to be a simple, efficient screening tool for NAFLD/MAFLD [25]. The following formula was used to calculate the HSI: HSI = 8 × ALT/AST ratio + BMI (+2 for diabetes; +2 if female) [25].

### 2.5. Subgroup Analysis

To evaluate the relationship of MAFLD with CSI across different types of EC, individuals were classified as type I EC and type II EC. Subsequently, we analyzed MAFLD-related indices and CSI in type I EC patients without T2DM.

### 2.6. Statistical Analysis

Statistical analyses were conducted using SPSS software (IBM Corp. Released 2019. IBM SPSS Statistics for Mac OS, Version 26.0. Armonk, NY, USA: IBM Corp.) and R statistical language (version 4.0.2, R Foundation for Statistical Computing, Vienna, Austria). The visualization was performed using the R package ggplot2. When the continuous variables satisfy the normal distribution, the mean ± standard deviation of the corresponding variable will be counted, and when the data do not satisfy the normal distribution, the median (upper and lower quartiles) of the corresponding variable will be counted. Categorical variables are expressed as frequencies and percentages. Student’s *t*-tests or Wilcoxon rank-sum tests were used to analyze the continuous variables. For comparisons between two groups that did not have a normal distribution, the non-parametric Wilcoxon rank-sum test was used. Categorical variables were analyzed by Chi-square tests or Fisher’s exact tests. Adjusted analyses were performed using multiple logistic regression to investigate binary associations. All covariates with a *p* value less than 0.20, based on univariate analysis, were included in the multivariate model. Multicollinearity was examined using the variance inflation factor (VIF). Survival curves with Kaplan–Meier estimates were used to compare overall survival between groups, with the comparison of survival curves by Log rank test/Cox regression analysis. Proportional risk hypothesis tests were performed using the survivor package and fitted survival regressions. The results were visualized using the survminer package as well as the ggplot2 package. Differences were considered statistically significant for *p* values < 0.05.

## 3. Results

### 3.1. Baseline Characteristics of the Study Subjects

The study included 725 EC participants who had abdominal ultrasound and biochemical data, among whom 201 (27.7%) had MAFLD and 524 (72.3%) had non-MAFLD (95% confidence interval (Cl) = 0.245–0.311). An overview of a participant’s baseline characteristics is given in Supplementary Table S1. There was a significant difference in the average age between the MAFLD and non-MAFLD groups (*p* = 0.015). Compared to the non-MAFLD group, the MAFLD group’s BMI was considerably higher (*p* < 0.001). The participants with MAFLD were older and more likely to be overweight/obese than that of the non-MAFLD group. However, statistically significant differences were observed only for CSI among factors associated with prognosis, including FIGO stage, histological grade, MI, LVSI, and LNM. (*p* = 0.027, Supplementary Table S1).

### 3.2. Survival Analyses

EC patients were divided into CSI (−) and CSI (+), and MAFLD (−) and MAFLD (+). To compare the overall survival of the two subgroups, Kaplan-Meier survival curves were calculated. Supplementary Figure S1 illustrates that EC patients with CSI significantly predicted a worse OS (*p* = 0.020). As shown in the picture, there was no significant difference in survival between patients with MAFLD and those without MAFLD (*p* = 0.952).

### 3.3. Independent Risk Factors for CSI in EC Patients

The expression of ER and PR was higher among EC patients with CSI (*p* < 0.001, Table 1). FIGO stage and histological grade were also associated with CSI (*p* < 0.001, Table 1). There was no significant difference between the two groups in serum fasting plasma glucose (FPG), triglycerides (TGs), high-density lipoprotein cholesterol (HDL) or aspartate aminotransferase (AST) (all *p* > 0.05, Table 2). Abdominal Ultrasound and ALT (alanine aminotransferase) significantly differed between the CSI (−) group and the CSI (+) group (*p* = 0.043 and *p* = 0.017, Table 2). The HSI between two subgroups was close to the threshold for the 0·05 significance level (*p* = 0.058, Table 2). However, the prevalence of MAFLD was significantly associated with CSI among EC patients in the univariate analysis (*p* = 0.027, Table 2).

**Figure 2:**
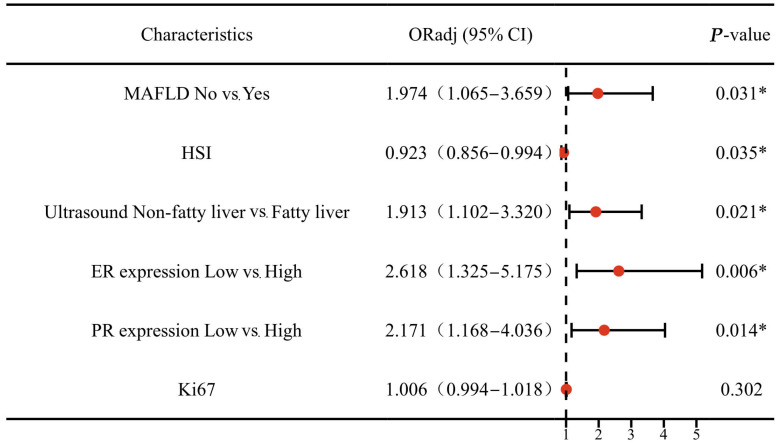
The Multivariable Logistic Regression Analysis for CSI among EC patients. Abbreviations: ER: estrogen receptor; PR: progesterone receptor; Ki67: proliferation cell nuclear antigen; FPG: fasting plasma glucose; TG: triglyceride; HDL: high-density lipoprotein cholesterol; ALT: alanine aminotransferase; AST: aspartate aminotransferase; HSI: hepatic steatosis index; MAFLD: metabolic dysfunction-associated fatty liver disease; EC: endometrial cancer; *p*-value: *p* value adjusted for age, BMI and histologic grade. * *p* ˂ 0.05 was considered to be statistically significant.

After adjusting for age, BMI and histologic grade, MAFLD was significantly associated with cervical stromal involvement in the total population (odds ratio (OR) 1.974; 95% confidence interval (CI) 1.065–3.659; *p* = 0.031; Figure 2).

### 3.4. Independent Risk Factors for CSI in Type I/Type II EC Patients

**Figure 3:**
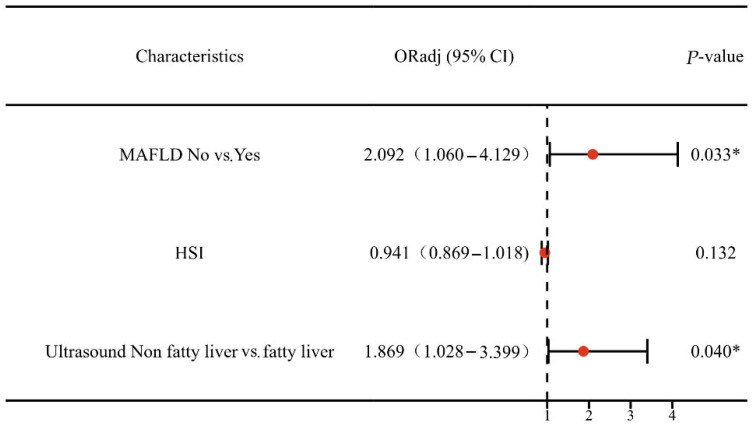
The Multivariate Logistic Regression Analysis for CSI among Type I EC Patients. Abbreviations: HSI: hepatic steatosis index; MAFLD: metabolic dysfunction-associated fatty liver disease; EC: endometrial cancer; *p*-value: *p* value adjusted for age, BMI and histologic grade. * *p* ˂ 0.05 was considered to be statistically significant.

After this, we categorized the participants into type I EC and type II EC groups for subgroup analysis based on their histological types and ER/PR expression status. Table 3 lists the metabolic indicators of the subjects with and without CSI among type I EC/type II EC). The type I EC group consisted of 605 participants in total, while the type II EC group contained 120 subjects. In the univariate analysis of the type I EC group, MAFLD was correlated with CSI (*p* = 0.022, Table 3). However, in the type II EC group, there was an absence of significant association between MAFLD and CSI (*p* = 0.838, Table 3). Among type I EC patients, HSI was the statistically significant difference between CSI (−) group and CSI (+) group (*p* = 0.049, Table 3). Although the difference was modest, it was statistically significant. We did not find any statistically significant difference between HSI and the risk of CSI in the type II EC group (*p* = 0.451, Table 3). Then, we analyzed six factors, including MAFLD, histologic grade, ER/PR expression, Ki67 index and HSI, in a stepwise manner. Remarkably, after adjusting for age, BMI and histologic grade, MAFLD remained the independent factor associated with cervical stromal involvement in the type I EC group (odds ratio (OR) 2.092; 95% confidence interval (CI) 1.060–4.129; *p* = 0.033; Figure 3).

### 3.5. Relationship between CSI and HSI in Type I EC Patients without T2DM

Type I EC and MAFLD were all closely correlated with T2DM. In order to further explore the clinical application potential of HSI, we concentrated more on type I EC patients without T2DM. It was found by logistic regression analysis that HSI was strongly linked with CSI in type I EC patients without T2DM (odds ratio (OR) 0.873; 95% confidence interval (CI) 0.778–0.979; *p* = 0.020; Table 4).

## 4. Discussion

In this study, we demonstrated that MAFLD is an independent factor associated with CSI in EC patients. In the type I EC group, there was a link between MAFLD and CSI, but in the type II EC group, there was no evidence of such an association. We further demonstrated that HSI may serve as a predictor of CSI in type I EC patients without T2DM.

Over the past decades, the prevalence of MAFLD/NAFLD has increased dramatically worldwide. MAFLD/NAFLD and some extrahepatic cancers (especially obesity-related tumors) have drawn considerable attention [30], such as colon cancer [31,32,33], breast cancer [34,35] and prostate cancer [36]. According to a recent study, women with EC have a significantly increased risk of developing NAFLD [37,38]. In this study, we evaluated 725 patients with newly diagnosed endometrial cancer who underwent bilateral salpingooophorectomy and total hysterectomy, either with or without lymphadenectomy. We found that the prevalence of MAFLD was 27.7% among these patients. Multiple studies have shown that conditions associated with disordered metabolism, such as obesity and hyperglycemia, may play a role in the development of endometrial cancer [39,40]. NAFLD is also closely associated with insulin resistance, metabolic syndrome, and diabetes mellitus. Considering these findings, it suggests that MAFLD/NALFD is considered one of the most common comorbidities in patients with EC and is a risk factor for the development of EC. However, these studies did not focus on prognostic factors in endometrial cancer but rather on the occurrence of endometrial cancer in general.

The clinical relevance of changing the name of NAFLD to MAFLD was covered in an international expert consensus statement on a new definition of MAFLD in early 2020, which also identified the disease’s heterogeneity and underlying metabolic variables as the key contributors to disease development [29]. MAFLD, encompassing clinical features, is a more appropriate nomenclature. MAFLD is usually a silent liver disease that does not cause any symptoms. However, MAFLD is involved in many conditions, including T2DM and obesity, and could also be a hepatic manifestation of MetS. These factors are also known as risks for EC.

CSI occurs in 5–10% of EC patients [41]. It is estimated that the 5-year overall survival drops to 75% when CSI is present, defined as FIGO stage II EC, compared to 88% for stage I [42]. CSI is reported by its ability to predict EC recurrence and plays important roles in the decision making of adjuvant therapy after surgery [9,31]. A previous study showed that CSI increased the rate of lymphatic metastasis to approximately 35–40% [43]. It has been reported that approximately 12% of patients with CSI will develop parametrial invasion [44]. While clinicians continue to overlook MAFLD in EC patients, there is growing awareness about its negative health effects. On the basis of our results, the relationship between MAFLD and CSI deserves further investigation to understand its potential impacts on endometrial cancer progression.

The potential mechanisms linking MAFLD to the CSI of endometrial cancer might be complicated and require further investigation. First, there is evidence that chronic systemic inflammation may increase the likelihood of EC development [45]. It is known that the oxidative and inflammatory environment in NAFLD is principally important in the promotion of tumorigenesis [46]. Intrahepatic lipid accumulation might trigger an inflammatory response by inducing the secretion of pro-inflammatory cytokines. [47,48]. Second, the liver plays a significant role in the regulation of energy metabolism. Some studies have shown that MAFLD/NAFLD patients have low levels of lipocalin and high levels of leptin. Adiponectin can block colon cancer cell growth via AMPc-activated protein kinase (AMPK) and induce caspase-dependent pathways leading to endothelial cell apoptosis. In colon cancer, leptin exerts pro-carcinogenic effects by the activation of the MAPK (mitogen-activated protein kinase) pathway [49]. Third, the insulin growth factor-1 (IGF-1) axis is activated by insulin resistance (IR) and create a microenvironment conducive to cancer development [50].

We also assessed the hormone receptor status of the EC cases in an effort to clarify the mechanistic relationship between MAFLD and endometrial cancer. In accordance with pathogenesis and biological behavior characteristics, type I EC and type II EC are the two categories into which EC is traditionally divided [7]. Endometrioid EC is driven by obesity and insulin resistance [51]. This may lead to MAFLD being more strongly associated with type I EC. There are several complex characteristics associated with endometrial carcinoma in patients with type 2 diabetes mellitus, which increase the difficulty of treatment in most cases [52]. We further evaluated the association of HSI and MAFLD with CSI in patients with type I EC without T2DM.

As the number of cancer patients with underlying fatty liver is increasing, we need an easy and feasible tool to screen this group of patients to treat them accurately. Individualized treatment of gynecologic malignancies is very necessary [53]. Another interesting finding was that the use of HSI may be useful for preoperative prediction of MAFLD and CSI in type I EC patients without T2DM.

There are several limitations to this study. First, it is important to note that this study was a retrospective study conducted by a single center, and there may be selection bias. The sample size was not calculated. Our limited sample size may have increased the risk of false negatives. Therefore, causative or temporal relationships between MAFLD and CSI of endometrial cancer could not be determined. Future studies should include a large, multicenter sample and more prospective experimental studies. Second, the diagnosis of MAFLD was based on ultrasonography without histological confirmation. Third, most of the subjects were women from Fujian, which may limit the generalizability of our work to other demographic groups. Fourth, several studies have demonstrated that the accurate way for determining the prognosis of EC patients is the study of molecular and genomic profiling [54]. Evaluating radiomics and radiogenomics signatures can help determine prognosis and plan the best treatment options for EC patients [55]. This also seems to be an interesting area worth pursuing in future research. Overall, the presence of MAFLD/NAFLD is an independent risk factor for CSI among EC patients. Additional studies will be needed to unveil the potential mechanisms underlying the association between MAFLD and EC.

## 5. Conclusions

In conclusion, our results demonstrated that the prevalence of MAFLD in patients with EC is high and that MAFLD was significantly associated with CSI in EC patients. Given these data, clinicians should pay more attention to EC patients with MAFLD. Early recognition and aggressive management of shared risk factors may improve the survival of oncology patients and reduce any delays in treatment. Therefore, screening and early diagnosis of MAFLD in patients with endometrial cancer may be important to establish timely intervention in this high-risk population. In addition, HSI is recommended as a simple non-invasive tool that can be used as part of a preoperative follow-up index for type I EC patients without T2DM.

## Figures and Tables

**Table 1:** Patient Demographics and Clinical Characteristics with CSI in EC Patients.

Characteristic	CSI (−)	CSI (+)	*p*
*n*	619	106	
Age, median (IQR)	54 (49, 59)	53.5 (49, 58.8)	0.612
BMI, median (IQR)	24.1 (22.2, 26.6)	23.7 (22.0, 26.3)	0.448
Menopause status, *n* (%)			0.265
Premenopausal	285 (46%)	55 (51.9%)	
Postmenopausal	334 (54%)	51 (48.1%)	
History of diabetes, *n* (%)			0.608
No	498 (80.5%)	83 (78.3%)	
Yes	121 (19.5%)	23 (21.7%)	
History of hypertension, *n* (%)			0.528
No	389 (62.8%)	83 (64.8%)	
Yes	230 (37.2%)	45 (35.2%)	
FIGO stage, *n* (%)			<0.001 *
I	576 (93.1%)	0 (0%)	
II–IV	43 (6.9%)	106 (100%)	
Histologic grade, *n* (%)			<0.001 *
G1	322 (52.0%)	29 (27.4%)	
G2	192 (31.0%)	40 (37.7%)	
G3	45 (7.3%)	17 (16.0%)	
ER expression, *n* (%)			<0.001 *
Low	57 (9.2%)	31 (29.2%)	
High	478 (77.2%)	67 (63.2%)	
PR expression, *n* (%)			<0.001 *
Low	89 (14.4%)	37 (34.9%)	
High	446 72.1%)	61 (57.5%)	
Ki67 %, median (IQR)	50 (30, 70)	40 (30, 60)	0.013 *

Note. Data are expressed as median (interquartile range (IQR)), or *n* (%). * *p* ˂ 0.05 was considered to be statistically significant. Abbreviations: EC: endometrial cancer; BMI: body mass index; CSI: cervical stromal involvement; ER: estrogen receptor; PR: progesterone receptor; Ki67: proliferation cell nuclear antigen. ER/PR low expression included ER/PR (−/±); ER/PR high expression included ER/PR (+/++/+++).

**Table 2:** Clinical Biochemical or Imaging Indicators with CSI among EC Patients.

Characteristic	CSI (−)	CSI (+)	*p*
*n*	619	106	
FPG, median (IQR)	5.23 (4.85, 5.85)	5.18 (4.80, 5.88)	0.586
TG, median (IQR)	1.36 (1.00, 1.93)	1.31 (0.95, 1.73)	0.455
HDL, median (IQR)	1.31 (1.13, 1.54)	1.3 (1.11, 1.53)	0.526
ALT, median (IQR)	19.60 (13.40, 27.54)	15.95 (12.40, 24.50)	0.017 *
AST, median (IQR)	18.80 (15.30, 24.00)	18.20 (14.25, 24.00)	0.360
HSI, median (IQR)	35.35 (31.69, 39.24)	34.18 (30.82, 37.79)	0.058
Ultrasound			0.043*
Non-fatty liver	399 (64.5%)	79 (74.5%)	
Fatty liver	220 (35.5%)	27 (25.5%)	
MAFLD, *n* (%)			0.027 *
Non-MAFLD	438 (70.8%)	86 (81.1%)	
MAFLD	181 (29.2%)	20 (18.9%)	

Note. Data are expressed as median (interquartile range (IQR)), or *n* (%). * *p* ˂ 0.05 was considered to be statistically significant. Abbreviations: FPG: fasting plasma glucose; TG: triglyceride; HDL: high-density lipoprotein cholesterol; ALT: alanine aminotransferase; AST: aspartate aminotransferase; HSI: hepatic steatosis index; MAFLD: metabolic dysfunction-associated fatty liver disease; EC: endometrial cancer.

**Table 3:** Association Between Clinical Biochemical or Imaging Indicators and CSI among Type I/Type II EC Patients.

Characteristic	Type I EC			Type II EC		
	CSI (−)	CSI (+)	*p*-Value	CSI (−)	CSI (+)	*p*-Value
*n*	532	73		87	33	
FPG	5.25 (4.85, 5.87)	5.08 (4.74, 5.96)	0.251	5.17 (4.82, 5.66)	5.26 (5.00, 5.78)	0.313
TG	1.40 (1.04, 1.96)	1.23 (0.91, 1.73)	0.139	1.10 (0.81, 1.80)	1.42 (1.08, 1.74)	0.062
HDL	1.31 (1.12, 1.54)	1.32 (1.20, 1.57)	0.362	1.39 ± 0.30	1.22 ± 0.32	0.010 *
ALT	19.73 (13.53, 27.85)	16.50 (12.94, 25.20)	0.088	18.00 (12.75, 26.00)	15.5 (11.26, 21.00)	0.210
AST	18.70 (15.20, 23.93)	18.50 (14.20, 24.00)	0.624	19.4 (16.15, 25.85)	17.50 (14.40, 22.00)	0.273
HSI	35.65 (31.97, 39.88)	33.66 (30.60, 38.40)	0.049 *	33.70 ± 4.72	34.42 ± 4.52	0.451
Ultrasound			0.047 *			0.779
Non-fatty liver	338 (63.5%)	55 (75.3%)		61 (70.1%)	24 (72.7%)	
Fatty liver	194 (36.5%)	18 (24.7%)		26 (29.9%)	9 (27.3%)	
MAFLD, *n* (%)			0.022 *			0.838
Non-MAFLD	368 (69.2%)	60 (82.2%)		70 (80.5%)	26 (78.8%)	
MAFLD	164 (30.8%)	13 (17.8%)		17 (19.5%)	7 (21.2%)	
Ki67	40 (20, 50)	40 (30, 60)	0.210	60 (40, 80)	55 (50, 72.5)	0.813

Note. Data are expressed as median (interquartile range (IQR)), mean ± SD, or *n* (%). * *p* ˂ 0.05 was considered to be statistically significant. Abbreviations: FPG: fasting plasma glucose; TG: triglyceride; HDL: high-density lipoprotein cholesterol; ALT: alanine aminotransferase; AST: aspartate aminotransferase; HSI: hepatic steatosis index; MAFLD: metabolic dysfunction-associated fatty liver disease; EC: endometrial cancer.

**Table 4:** Univariate and Multivariate Logistic Regression Analysis of Independent Risk Factors for CSI in Type I EC Patients without T2DM.

Characteristics	Total (N)	Univariate Analysis		Multivariate Analysis	
		Odds Ratio (95% CI)	*p*-Value	Odds Ratio (95% CI)	*p*-Value
MAFLD	484				
Non-MAFLD	362	Reference		Reference	
MAFLD	122	2.572 (1.752–3.392)	0.024 *	2.718 (1.089–6.783)	0.032 *
HSI	484	1.079 (1.020–1.139)	0.012 *	0.873 (0.778–0.979)	0.020 *
Ultrasound, *n* (%)	484				
Non-fatty liver	327	Reference			
Fatty liver	157	1.879 (1.210–2.548)	0.065		
Age	484	1.009 (0.976–1.041)	0.599		
BMI	484	1.058 (0.973–1.143)	0.190		
Histologic grade	468				
G1	170	Reference			
G2	298	2.251 (1.670–2.833)	0.006 *		

Note. * *p* ˂ 0.05 was considered to be statistically significant. Abbreviations: EC: endometrial cancer; BMI: body mass index; CSI: cervical stromal involvement; HSI: hepatic steatosis index; MAFLD: metabolic dysfunction-associated fatty liver disease; *p*-value: *p* value adjusted for age, BMI and histologic grade.

## Data Availability

The data presented in this study are available on request from the corresponding author. The data are not publicly available due to privacy.

## Supplementary Materials

The following supporting information can be downloaded at https://www.mdpi.com/article/10.3390/curroncol30040287/s1, Figure S1: a. Kaplan-Meier (KM) survival curves of CSI (-) and CSI (+) groups. b. Kaplan-Meier (KM) survival curves of MAFLD (-) and MAFLD(+) groups; Table S1: Comparison of the Demographic and Clinical Characteristics among EC patients with and without MAFLD.
