# Supplementary material for: Metabolic Dysfunction-Associated Fatty Liver Disease (MAFLD) Is Associated with Cervical Stromal Involvement in Endometrial Cancer Patients: A Cross-Sectional Study in South China
Source: Curr Oncol. 2023 Mar 29;30(4):3787–99. doi: 10.3390/curroncol30040287 (PMC10136854; doi:10.3390/curroncol30040287)
Supplement: Supplementary file 1 [file curroncol-30-00287-s001.zip › curroncol-2104232-supplementary.pdf]

Current Oncology

MDPI

Supplementals

Metabolic Dysfunction-Associated Fatty Liver Disease (MAFLD) Is Associated with Cervical Stromal Involvement in Endometrial Cancer Patients: A Cross-Sectional Study in South China

Xite Lin [1,2,3,†], Chunxia Chen [4,†], Tingting Jiang [1,2,3], Jincheng Ma [1,2,3], Lixiang Huang [1,2,3], Leyi Huang [1,2,3], Huifang Lei [1], Yao Tong [1], Guanxiang Huang [1,2,3], Xiaodan Mao [1,2,3,*] and Pengming Sun [1,2,3,*]

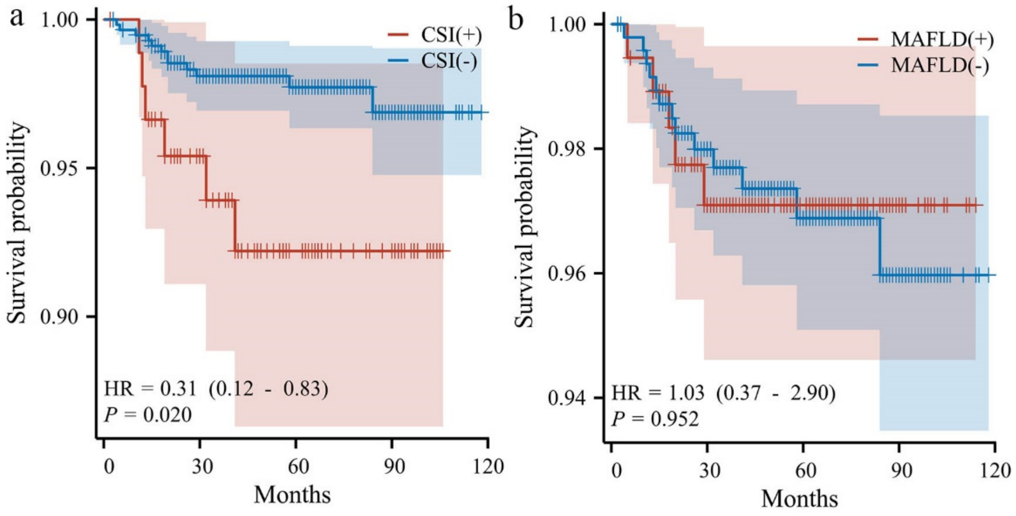

Figure S1. a. Kaplan-Meier (KM) survival curves of CSI (-) and CSI (+) groups. b. Kaplan-Meier (KM) survival curves of MAFLD (-) and MAFLD(+) groups.

Table S1. Comparison of the demographic and clinical characteristics among Endometrial cancer patients with and without MAFLD.

Characteristic	Non-MAFLD	MAFLD	P
n	524	201	
Age, median (IQR)	53 (48, 59)	55 (50, 60)	0.015*
BMI, median (IQR)	23.4 (21.6, 25.9)	25.9 (23.9, 28.0)	< 0.001*
Menopause status, n (%)			0.834
Premenopausal	247 (47.1%)	93 (46.3%)	
Postmenopausal	277 (52.9%)	108 (53.7%)	
FIGO stage, n (%)			0.056
I	117 (22.3%)	32 (15.9%)	
II-IV	407 (77.7%)	169 (84.1%)	
Histologic grade, n (%)			0.367
G1	248 (47.3%)	103 (51.2%)	
G2	163 (31.1%)	69 (34.3%)	
G3	49 (9.4%)	13 (6.5%)	
CSI, n (%)			0.027*
No	86 (16.4%)	20 (10%)	
Yes	438 (83.6%)	181 (90%)	
LVSI, n (%)			0.243

Characteristic	Non-MAFLD	MAFLD	P
No	470 (89.7%)	186 (92.5%)	
Yes	54 (10.3%)	15 (7.5%)	
MI, n (%)			0.449
<1/2	410 (78.2%)	152 (75.6%)	
≧1/2	114 (21.8%)	49 (24.4%)	
LNM, n (%)			0.520
No	270 (51.5%)	115 (57.2%)	
Yes	30 (5.7%)	10 (5.0%)	

Note. Data are expressed as median (interquartile range (IQR)), or n (%). * $p < 0.05$ was considered to be statistically significant. Abbreviations: MAFLD: metabolic dysfunction-associated fatty liver disease; EC: endometrial cancer; BMI: body mass index; CSI: cervical stromal involvement; LVSI: lymph-vascular space invasion; MI: myometrial invasion; LNM: lymph node metastasis.
